# Supplementary material for: Complex‐centric proteome profiling by SEC‐SWATH‐MS
Source: Mol Syst Biol. 2019 Jan 14;15(1):e8438. doi: 10.15252/msb.20188438 (PMC6346213; doi:10.15252/msb.20188438)
Supplement: Supplementary file 7 — Dataset EV6 [file MSB-15-e8438-s007.zip › feature_plots_bioplex/Q9NUL3.pdf]

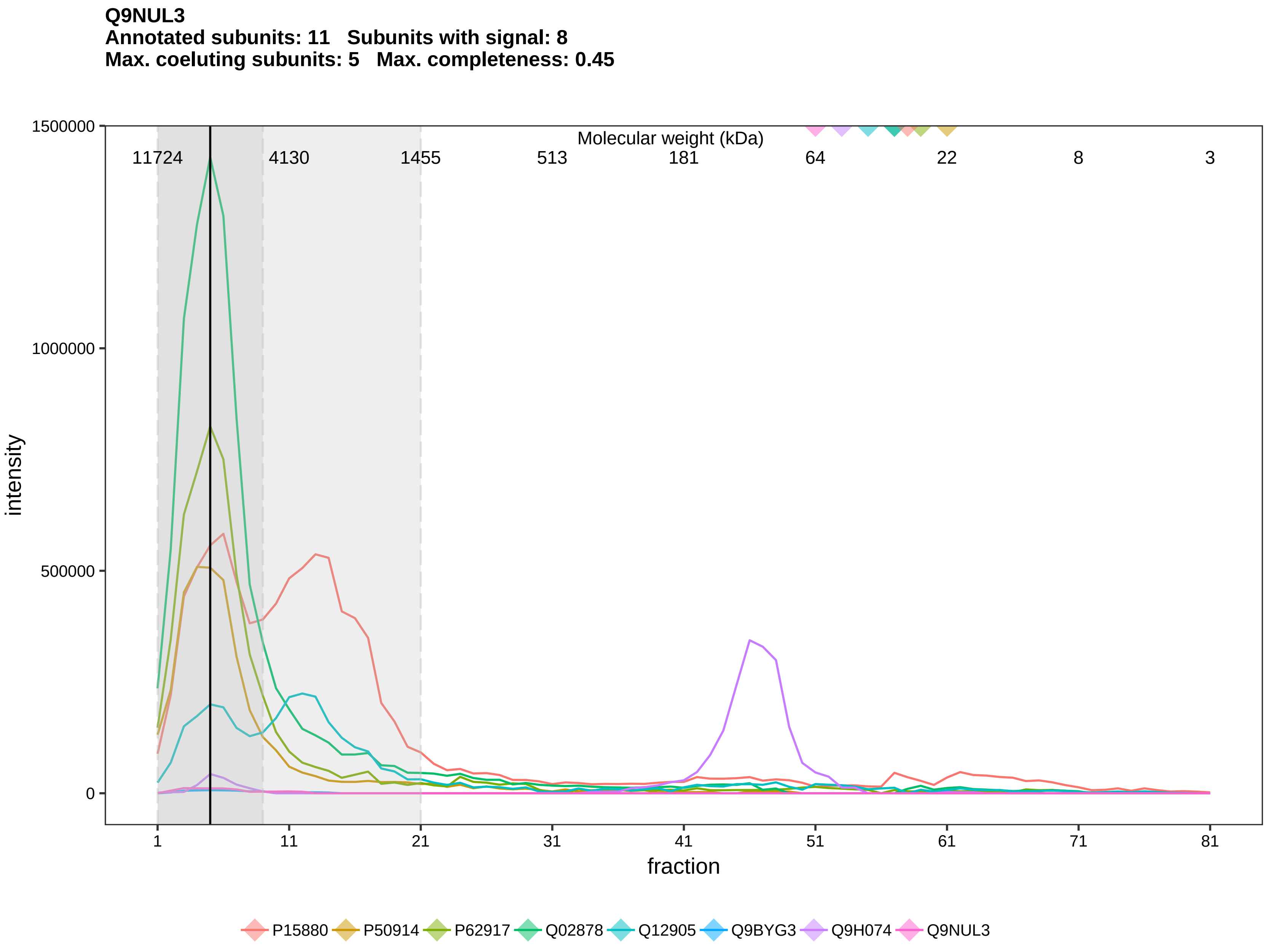
Q9NUL3
Annotated subunits: 11 Subunits with signal: 8
Max. coeluting subunits: 5 Max. completeness: 0.45
Molecular weight (kDa)
11724
4130
1455
513
181
64
22
8
3
1500000
1000000
500000
0
intensity
1
11
21
31
41
51
61
71
81
fraction
P15880
P50914
P62917
Q02878
Q12905
Q9BYG3
Q9H074
Q9NUL3
